# Effect of concurrent action observation, peripheral nerve stimulation and motor imagery on dexterity in patients after stroke: a pilot study

**DOI:** 10.1038/s41598-024-65911-7

**Published:** 2024-06-27

**Authors:** Sarina Seitz, Corina Schuster-Amft, Jasmin Wandel, Leo H. Bonati, Katrin Parmar, Hans Ulrich Gerth, Frank Behrendt

**Affiliations:** 1 Research Department, Reha Rheinfelden, Rheinfelden, Switzerland; 2 Institute of Physiotherapy, Zurich University of Applied Sciences, Winterthur, Switzerland; 3 Department of Sport, Exercise and Health, University of Basel, Basel, Switzerland; 4 School of Engineering and Computer Science, Bern University of Applied Sciences, Biel, Switzerland; 5 Institute for Optimization and Data Analysis, Bern University of Applied Sciences, Biel, Switzerland; 6 Department of Neurology, University Hospital Basel, Basel, Switzerland; 7 Department of Clinical Research, University of Basel, Basel, Switzerland; 8 Department of Medicine, University Hospital Münster, Münster, Germany

**Keywords:** Medical research, Motor control

## Abstract

Research to improve and expand treatment options for motor impairment after stroke remains an important issue in rehabilitation as the reduced ability to move affected limbs is still a limiting factor in the selection of training content for stroke patients. The combination of action observation and peripheral nerve stimulation is a promising method for inducing increased excitability and plasticity in the primary motor cortex of healthy subjects. In addition, as reported in the literature, the use of action observation and motor imagery in conjunction has an advantage over the use of one or the other alone in terms of the activation of motor-related brain regions. The aim of the pilot study was thus to combine these findings into a multimodal approach and to evaluate the potential impact of the concurrent application of the three methods on dexterity in stroke patients. The paradigm developed accordingly was tested with 10 subacute patients, in whom hand dexterity, thumb-index pinch force and thumb tapping speed were measured for a baseline assessment and directly before and after the single intervention. During the 10-min session, patients were instructed to watch a repetitive thumb-index finger tapping movement displayed on a monitor and to imagine the sensations that would arise from physically performing the same motion. They were also repeatedly electrically stimulated at the wrist on the motorically more affected body side and asked to place their hand behind the monitor for the duration of the session to support integration of the displayed hand into their own body schema. The data provide a first indication of a possible immediate effect of a single application of this procedure on the dexterity in patients after stroke.

## Introduction

Stroke is one of the most common causes of disability in adults^1^, resulting in a significant need for medical rehabilitation. This remains an important issue in rehabilitation research^2^ although substantial efforts have been devoted to improve functional recovery^3,4^. A number of randomized-controlled studies and experimental trials on the effectiveness, for instance of virtual reality rehabilitation, with promising results have been found^5^. A major impairment after a stroke can be the loss of dexterity as upper limb skills are essential in everyday activities that require fine movements for handling and manipulating a variety of objects. The consequences of a persistent, non-recovered upper limb function impairment are immense and often directly lead to a reduced quality of life^6^. In the attempt to foster recovery, the measures taken to support motor improvement depend on a number of central nervous system processes referred to as neuroplasticity^7^.

Two well established interventions to support neuroplastic changes in patients with a limited capacity to physically practice are action observation and motor imagery^8^. For upper limb rehabilitation after stroke, action observation (AO) was found beneficial in improving motor function and dependence in activities of daily living but its clinical relevance is unclear^9^. AO is a cognitive process involving the perception and comprehension of an action performed by another individual. It typically involves visually observing someone else's actions, which can activate similar neural networks in the observer's brain as if they were performing the action themselves^10^. On the other hand, Motor Imagery (MI) is a cognitive process in which an individual mentally simulates or rehearses a specific motor action without physically executing it^11^. During MI, individuals vividly imagine themselves performing a movement, engaging sensory and motor representations in the brain. When added to rehabilitation interventions, it can result in improvements in upper limb function and movement, while there is no evidence for a beneficial effect of MI alone compared to conventional treatment^12^. However, evidence exists of plastic changes alongside behavioral improvements during MI^13^.

In neurophysiological research using Electroencephalography (EEG) or Functional magnetic resonance imaging (fMRI), it was found that the combined application of AO and MI (AOMI) can increase brain activity involving cortical areas more comprehensively compared to applying them independently. Neuroimaging studies on action observation combined with motor imagery (AOMI) suggest more robust activation of motor-related brain regions compared to conditions involving only AO or only MI, as demonstrated using fMRI^14–17^, EEG^18–20^, Transcranial magnetic stimulation^21–24^ or Functional near-infrared spectroscopy^25,26^. For both, a shared neural network was proposed, but with differences in the brain activity overlap compared with physically performed movements^27^. AOMI was therefore suggested to potentially entail a wider overlap with motor execution^28^.

There are several available studies that have focused on the immediate effects of synchronous AO + MI instructions on neurophysiological and behavioural parameters for reviews, see Eaves et al., 2016a; Emerson et al., 2018; McNeill et al., 2020; Vogt et al., 2013; Wright et al., 2021). A recent paper reported two meta-analyses to quantify changes in corticospinal excitability and motor skill performance during combined, simultaneous AOMI compared to AO, MI and control conditions supporting the effectiveness of AOMI as an alternative intervention to AO and MI^28^. Based on these neurophysiological and behavioral findings, AOMI was accordingly suggested to be a beneficial approach for motor rehabilitation purposes^29^. In stroke recovery, a few studies reported on improvements in motor performance and cortico-motor involvement measures in patients who received AOMI^30–32^. Choi et al. found a positive effect in both corticospinal excitability and upper-limb function in stroke patients in a randomized controlled trial^32^ and, in terms of the upper-limb function measured using the Fugl-Meyer Assessment, improvements were also reported by Robinson-Bert and Woods^30^. Clinically relevant benefits of synchronous AOMI treatment in a group of stroke survivors compared with asynchronous AO and MI treatment highlight the potential benefit of synchronous AOMI practice^31^. For an overview of the variety of AOMI practices, see Eaves et al.^29^.

Another method to achieve an enhanced facilitatory effect of AO is to combine it with peripheral nerve stimulation (PNS). In two studies with healthy participants, a stimulation protocol was used that consisted of a combination of watching a video showing repetitive thumb-index tapping movements and concurrent PNS^33,34^. The concurrent AO-PNS was found to induce plasticity in the primary motor cortex, which only occurred in combination but not when applied alone. The effect was remarkably present for a duration of at least 45 min after a single, brief intervention^33^.

Based on these findings, it seemed worthwhile to collect first data on the impact of the combination of the described intervention options with stroke patients. Therefore, the aim of the study was to assess whether a combined, concurrent application of Action Observation, Motor Imagery and Peripheral Nerve Stimulation would result in a measurable improvement in dexterity using functional tests in patients after stroke.

## Methods

### Patients

Patients after their first ischemic or hemorrhagic stroke, who were able to sit independently, scored higher than 19 in the Montreal Cognitive Assessment^35^ and higher than 1 in the Box-and-Block Test^36^ could be enrolled in the study. Since the level of neuroplasticity and thus the recovery-related processes post-stroke are time-dependent^37,38^, patients were included if they were still in the pre-chronic phase (< 6 months). The patients further needed to satisfactorily score in two out of three of the following motor imagery ability instruments: (a) Kinesthetic and Visual Imagery Questionnaire score of 30/50^39^, (b) Mental rotation > 75%^40^, and (c) Mental chronometry ratio of 1 ± 0.25^39^. Exclusion criteria were visual impairment, epileptic seizures in the past six months, other neurological, metabolic, or mental disorders, a pacemaker, or a metal implant in the hand or forearm of the affected side. The study protocol was approved by the local ethics committee for Northwest and Central Switzerland (Project-ID: 2022-00815) and conformed to the declaration of Helsinki. All patients provided written informed consent before the start of the data collection.

### Intervention procedure

**Figure 1:**
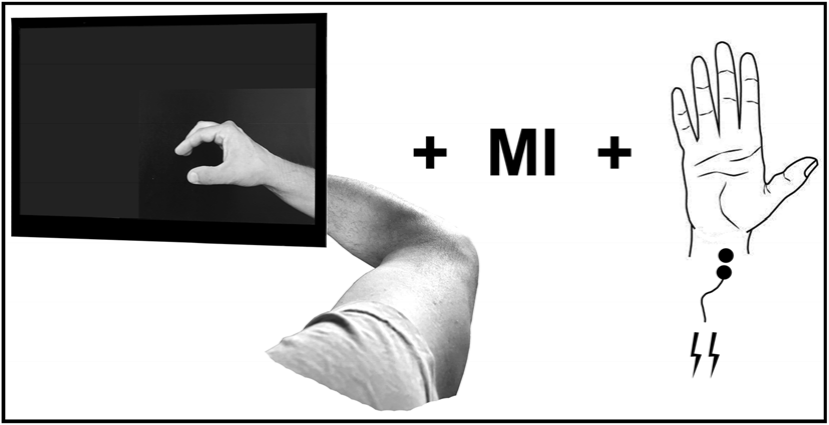
Experimental set-up representing the concurrent use of action observation, motor imagery and peripheral nerve stimulation at the wrist.

The patients were requested to sit relaxed and observe a computer screen showing a lower arm and hand performing repetitive thumb-index finger-tapping movements. Corresponding to the affected side of the body, the right- or left-hand video was selected. The patients were also asked to place their affected hand behind the screen so that the depicted hand and part of the forearm on the screen would appear to be their own (Fig. 1). This was done to facilitate the incorporation of the virtual hand into the individual's body schema^41^, which is the brain's representation of the body^42^, by closely matching the spatial orientation of their own hand. Regarding this part of the experimental approach, it must be noted that there is still limited literature concerning the effects of stroke on embodiment. Studies on this topic have uncovered both similarities and differences in the experiences of stroke survivors and healthy individuals. Borrego et al. found that both groups experienced a sense of embodiment and presence in a virtual environment, although to a lesser extent in stroke survivors^43^. Slightly deviating from that, using the well-established Rubber Hand Illusion paradigm, researchers found an even significantly stronger sense of body ownership and agency in patients compared to healthy individuals after stroke^44^. Here, the authors suggested that an increased dominance of visual input over proprioception may have contributed to the observed results^44^. However, using this approach seemed worthwhile for the current experiment. The patients were also instructed not to perform the observed movement themselves.

Patients watched a video sequence showing continuous, repetitive thumb-index tapping of either the right or left hand, depending on the impaired side of each participant, with the affected hand positioned behind the monitor (Fig. 1). On the screen, only a hand and part of the forearm were visible against a dark background. The experimental procedure was conducted in a darkened room, so that participants could only see and better concentrate on what was shown on the screen. They were asked to concurrently imagine the sensations of the thumb and index finger that would occur during the execution of the movement without actually performing it, which corresponds to the kinesthetic mode. The intervention/the video lasted for 10 min with the depicted tapping movement executed at a frequency of 2 Hz^33^. Also concurrently, while observing the movement, electrical stimuli were applied to the median nerve at the wrist. These stimuli were triggered approximately every 4 s at the end of a closing phase of thumb-index tapping movement, corresponding to every 8th thumb-index finger-tapping movement. The closing phase was chosen according to Bisio et al., who reported a higher excitability in the primary motor cortex during AO in this phase^33^. Furthermore, a nearly identical approach was chosen to ensure that the study participants were indeed attentive to the screen and did not shift their focus elsewhere. Thus, to enhance attention to the visual stimuli, the video was interrupted five times, each time displaying a black screen with a white cross for 5 s. The participants were instructed beforehand to count the number of occurrences.

Electrical stimuli were applied through a bipolar electrode connected to a Digitimer (DS7A, Welwyn Garden City, UK) constant current stimulator, using square wave pulses (duration 1 ms) at an intensity of three times the perceptual threshold, able to evoke a small twitch in the abductor pollicis brevis muscle which was tolerated by the patients. The presentation of the video sequence including the accurate timing of the trigger signals for the electrical stimulation was created using Psychopy3^45^.

The intervention was carried out once per patient, while dexterity of the affected side was assessed three times: 3–7 days before the intervention (baseline), and immediately before (pre) and after (post). For this, the following three different assessments were used at each of the three time points. (1) Gross manual dexterity: Box-and-Block Test^36^ performed once each time, (2) Thumb-index pinch force performed three times at each time point to determine the respective average. We used a hydraulic pinch gauge (North Coast Medical Inc., Morgan Hill, CA) to measure the maximum force between the thumb and index finger of the affected hand. (3) Thumb tapping speed: A hand held counter was used to determine the number of thumb movements within 10 s. This was also performed three times at each time point to obtain the mean values of the maximum possible tapping speed.

We conducted one-tailed paired t-tests on the different assessments results, but did not perform an additional analysis given the limited data. Due to the exploratory nature of the study, no adjustment of the alpha-level was conducted. Furthermore, to assess the strength of the results, we carried out a post-hoc power analysis.

## Results

**Figure 2:**
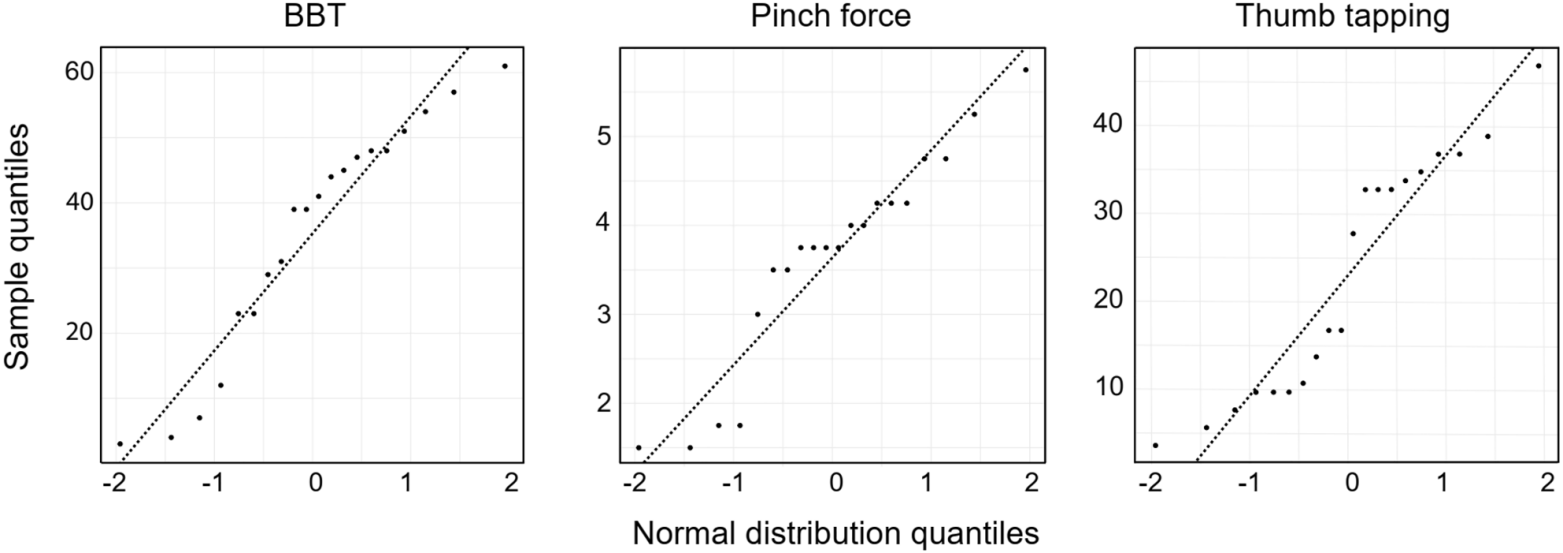
QQ-plots.

In total, ten patients (mean age of 63.4 ± 15.5 years, see Table 1 for all characteristics) could be included. Separate QQ-plots (Fig. 2) of all three data sets were used to assess normality. Despite slight deviations, they indicated a tendency for the data to follow a normal distribution.

**Table 1:** Patient characteristics.

ID	Gender	Age range	Month post-stroke	Type and site of lesion	MoCA	MC	MR	KVIQ-10
1	f	85–89	4	I, right, multilocular	22	0.52	25	25
2	m	55–59	1	I, brainstem	21	1.02	32	47
3	f	50–54	1	H, right BG	28	0.76	20	40
4	f	85–89	0.75	I, left middle CR	25	0.84	17	50
5	m	60–65	0.75	I, left middle CR	22	0.35	24	49
6	f	55–59	2	I, left middle CR	25	1.10	27	48
7	m	35–39	3.5	H, left BG	21	0.98	31	48
8	f	60–64	0.75	I, left basilar artery	28	1.03	20	34
9	f	70–74	5.25	I, left lenticulostriate artery	24	1.11	26	42
10	f	60–64	0.75	I, left anterior thalamus	22	0.74	32	46

*BG* basal ganglia, *CR* cerebral artery, *H* hemorrhagic, *I* ischemic, *KVIQ-10* Kinesthetic and Visual Imagery Questionnaire, *MC* mental chronometry, *MoCa* Montreal Cognitive Assessment, *MR* mental rotation.

**Figure 3:**
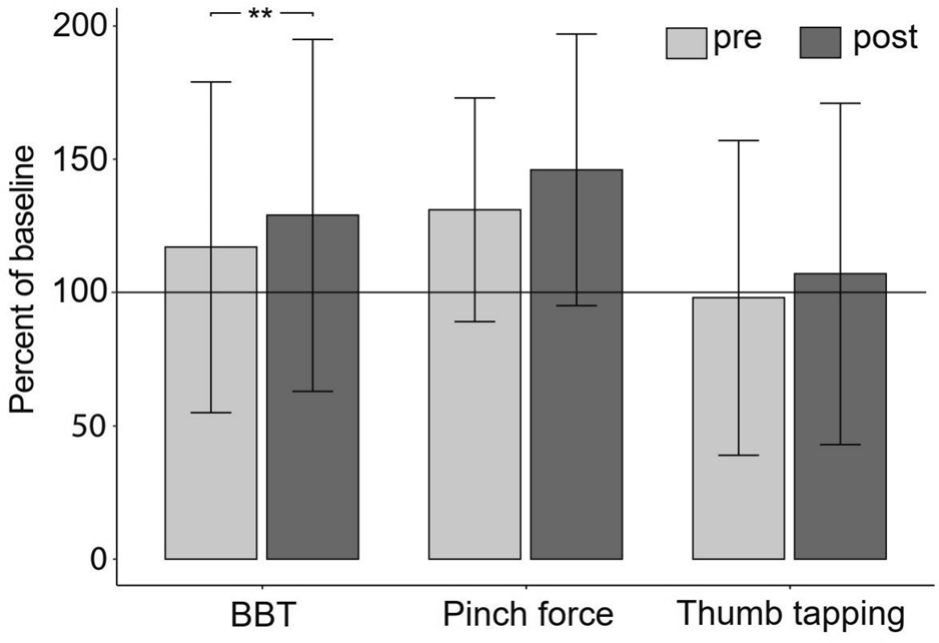
Mean (SD) scores directly before and after the intervention normalized to the respective baseline values measured 3–7 days before the intervention (100% line). ** P < 0.01. *BBT* Box-and-Block Test.

The one-tailed paired t-test showed that the difference (Fig. 3) between pre (mean 33.6 blocks, SD = 17.8) and post (mean = 37.0, SD = 19.0) BBT results was significant (t(9) = − 3.9, P = 0.002) with an effect size of Cohen's d = 1.23. For the pinch force assessment, data revealed no significant difference (t(9) = − 1.53, P = 0.08) between pre (mean = 3.4 kg, SD = 1.1) and post (mean = 3.8, SD = 1.3) with d = 0.48. This was also the case for the data from the thumb-tapping tests (t(9) = − 1.66, P = 0.06) between pre (mean = 22.2 repetitions, SD = 13.3) and post (mean = 24.1, SD = 14.4) with d = 0.53.

The post-hoc power analysis unveiled that the attained statistical power for the BBT data was 0.84. For the pinch force data and thumb tapping data, the statistical power was 0.27 and 0.30, respectively.

## Discussion

The aim of the present study was to investigate the potential effect of a combined application of action observation, motor imagery and electrical peripheral nerve stimulation on dexterity in patients after their first-ever stroke. The used paradigm extended the AO-PNS approach of Bisio et al. with healthy individuals, which showed a positive effect on corticospinal excitability^33^. We attempted to potentially enhance this effect by adding two further components. (1) The experimental setup was modified to facilitate the integration of the observed hand into the patient's individual body schema. (2) The patients were asked to imagine how the observed thumb-index movements would feel.

We found that a brief AOMI-PNS intervention may have induced a transient improvement in dexterity in the affected hand of the included patients. However, data also suggest that a single AOMI-PNS session is at least partly not sufficient to clearly improve the scores of the assessments used.

In this preliminary study, only a limited sample size was used and no control group included, which limits the interpretability of the data. Further, the possibility of a short-term learning effect cannot completely be ruled out, although we made efforts to mitigate its influence by adjusting the methodology accordingly. The thumb tapping speed test and pinch force test were executed six times each prior to the intervention event, and the final three were utilized to calculate the pre-intervention average. This procedure presumably did not entirely eradicate the learning effect, but it certainly minimized its impact. Further, on occasion, study patients reported experiencing minor motor fatigue as a result of the assessments conducted immediately before the intervention and also of the PNS itself, which might even have a reduction effect on the scores of the post measurements and counteract possible gains.

As we did not apply TMS and investigated a different population, it cannot necessarily be assumed that the stimulation protocol (AOMI-PNS) applied in this study also resulted in an increase of the M1 excitability as reported by Bisio et al. using AO-PNS^33^. The latter did not test for dexterity changes, however, evidence of a connection between increased M1 excitability and improved manual dexterity can be found in the literature^46,47^. Sun et al. found clinically relevant improvements in chronic stroke patients both in measures of motor performance and in cortico-motor involvement following synchronous AOMI treatment alongside physical rehabilitation^31^. In these patients, the synchronous AOMI treatment obviously had a distinct advantage over asynchronous AOMI. Capozio and colleagues, on the other hand, could not find an improvement in dexterity after a combined application of transcutaneous electrical stimulation of the spinal cord and MI^48^. However, they reported an acute effect of both applied in conjunction on cortical neural excitability assessed using transcranial magnetic stimulation.

Indeed, it could be argued that the requirements for the simultaneous and coordinated performance of AO and MI tasks might be too demanding from a cognitive point of view for some patients after a stroke. In order to avoid a bias due to cognitive overload, we carefully tested patients prior to study inclusion by using the Montreal Cognitive Assessment and three different assessments to evaluate their individual MI ability. Testing for an adequate MI performance was also applied by Sun et al., who at least used the short version of kinesthetic and visual imagery questionnaire^31^. All patients tested in the current study prior to a possible enrolment met the cognitive requirements.

In light of the results, the partially low statistical power and the existing literature, a next step would be to apply this intervention in an expanded cohort of stroke patients including a control group and an additional assessment of the neural activity at cortical level.

## Conclusion

In conclusion, this study demonstrates that applying the combination of the different described therapeutic approaches is possible with patients after stroke and provides an initial indication of a potential positive effect on dexterity.

## Data Availability

The dataset used and analyzed during the current study is available from the corresponding author upon reasonable request.
